# Thioredoxin-interacting protein: A new therapeutic target in bone metabolism disorders?

**DOI:** 10.3389/fimmu.2022.955128

**Published:** 2022-08-17

**Authors:** Na Jiang, Jinjin Liu, Conghui Guan, Chengxu Ma, Jinyang An, Xulei Tang

**Affiliations:** ^1^ The First Clinical Medical College of Lanzhou University, Lanzhou, China; ^2^ Department of Endocrinology, The First Hospital of Lanzhou University, Lanzhou, China

**Keywords:** Thioredoxin-interacting protein, osteoporosis, osteoarthritis, rheumatoid arthritis, osteoblasts, osteoclasts, chondrocytes

## Abstract

Target identification is essential for developing novel therapeutic strategies in diseases. Thioredoxin-interacting protein (TXNIP), also known as thioredoxin-binding protein-2, is a member of the α-arrestin protein family and is regulated by several cellular stress factors. TXNIP overexpression coupled with thioredoxin inhibits its antioxidant functions, thereby increasing oxidative stress. TXNIP is directly involved in inflammatory activation by interacting with Nod-like receptor protein 3 inflammasome. Bone metabolic disorders are associated with aging, oxidative stress, and inflammation. They are characterized by an imbalance between bone formation involving osteoblasts and bone resorption by osteoclasts, and by chondrocyte destruction. The role of TXNIP in bone metabolic diseases has been extensively investigated. Here, we discuss the roles of TXNIP in the regulatory mechanisms of transcription and protein levels and summarize its involvement in bone metabolic disorders such as osteoporosis, osteoarthritis, and rheumatoid arthritis. TXNIP is expressed in osteoblasts, osteoclasts, and chondrocytes and affects the differentiation and functioning of skeletal cells through both redox-dependent and -independent regulatory mechanisms. Therefore, TXNIP is a potential regulatory and functional factor in bone metabolism and a possible new target for the treatment of bone metabolism-related diseases.

## 1 Introduction

Bones provide structural support to the body and regulate the hematopoietic system. Bone metabolism involves two important processes: bone modeling and bone remodeling. Bone modeling is initiated during fetal development and continues until bone maturity is reached. Bone shape and structure are optimized by various factors and mechanical forces during development (1). Bone modeling helps to prevent bone damage by adjusting the structure to adapt to external loads (2). Conversely, bone remodeling maintains the stability of bone biomechanics, an important physiological bone renewal process, and includes the quiescent, activation, resorption, formation, and mineralization stages (3). Bone renewal is accomplished by a multicellular unit known as the bone remodeling unit, which includes four types of cells—bone lining cells, osteocytes, osteoclasts (OCs), and osteoblasts (OBs) (4). Bone development and growth involve chondrocytes (CCs), which do not function in isolation but are rather coordinated in the bone microenvironment (5). Bone remodeling depends on the coupling between bone formation by OBs and resorption by OCs (6). In the bone remodeling unit, bones can disappear owing to OC absorption and be replaced by OBs that synthesize the bone. Bone reconstruction and balance allow for continuous bone renewal (7), which can be disrupted by conditions such as aging and decreased estrogen levels. When bone resorption exceeds bone formation, it leads to bone loss and the destruction of bone structure and homeostasis, ultimately leading to various acquired metabolic bone diseases, such as osteoporosis (OP) (1, 2, 7). Oxidative stress is involved in bone metabolic changes and leads to high reactive oxygen species (ROS) levels. Excessive ROS activity and defects in the cellular antioxidant system lead to cellular redox imbalances and abnormal cellular bone metabolism (8). In addition, chronic inflammation can disrupt bone metabolism; cytokines activated during the inflammatory response can cause inflammation-related bone loss, including osteoarthritis (OA) and rheumatoid arthritis (RA) (9). In OA, CCs and synovial cells overproduce various inflammatory cytokines, such as interleukin (IL)-6, IL-1β, and tumor necrosis factor α (TNF-α), all of which are involved in immune response and mediate cartilage destruction (10). In RA, mononuclear macrophages and lymphocytes release numerous inflammatory factors, such as IL-1, IL-6, IL-17, TNF-α, and matrix metalloproteinases (MMPs), thereby inducing inflammatory reactions and leading to joint injury (11). OA, RA, and OP are the most common chronic diseases in the elderly population and significantly affect quality of life and life expectancy. They share similar pathophysiological pathways, including increased bone remodeling/absorption, age-related phenotypes, and the accumulation of inflammatory factors in the joints and bone tissues. The currently available treatments remain unsatisfactory; therefore, to develop more effective treatments, it is necessary to determine the key pathological targets responsible for promoting these diseases.

Thioredoxin-interacting protein (TXNIP) is involved in the intracellular redox system and is an essential mediator of oxidative stress and inflammatory response (12). TXNIP is referred to as a thioredoxin (TRX)-interacting protein because it interacts with TRX, an important intracellular antioxidant protein. TXNIP is one of several α-arrestin proteins involved in various important cellular processes, such as cell metabolism, inflammation, and cell death through redox-dependent and -independent pathways. TXNIP has attracted significant research interest owing to its involvement in glucose homeostasis, cancer (13), and neurodegenerative diseases (14). TXNIP has also been investigated in the context of the pathogenesis of various bone metabolism-related diseases. In this review, we discuss the structure and function of TXNIP, especially its expression in bone metabolic diseases and bone tissues and its involvement in the pathological processes associated with bone metabolic abnormalities, including the regulatory mechanism of TXNIP inflammation, oxidative stress, and autophagy in different types of bone cells. Furthermore, we provide evidence for the pathological contribution of oxidative stress and inflammation induced by different stress factors in OP, OA, and RA and their association with TXNIP. Finally, we summarize the application of TXNIP regulators in bone metabolism-related diseases to further clarify the mechanism underlying TXNIP involvement in bone metabolism and outline the provision of targets for the prevention and treatment of bone metabolism-related diseases.

## 2 TXNIP

### 2.1 Gene regulation of TXNIP

**Figure 1:**
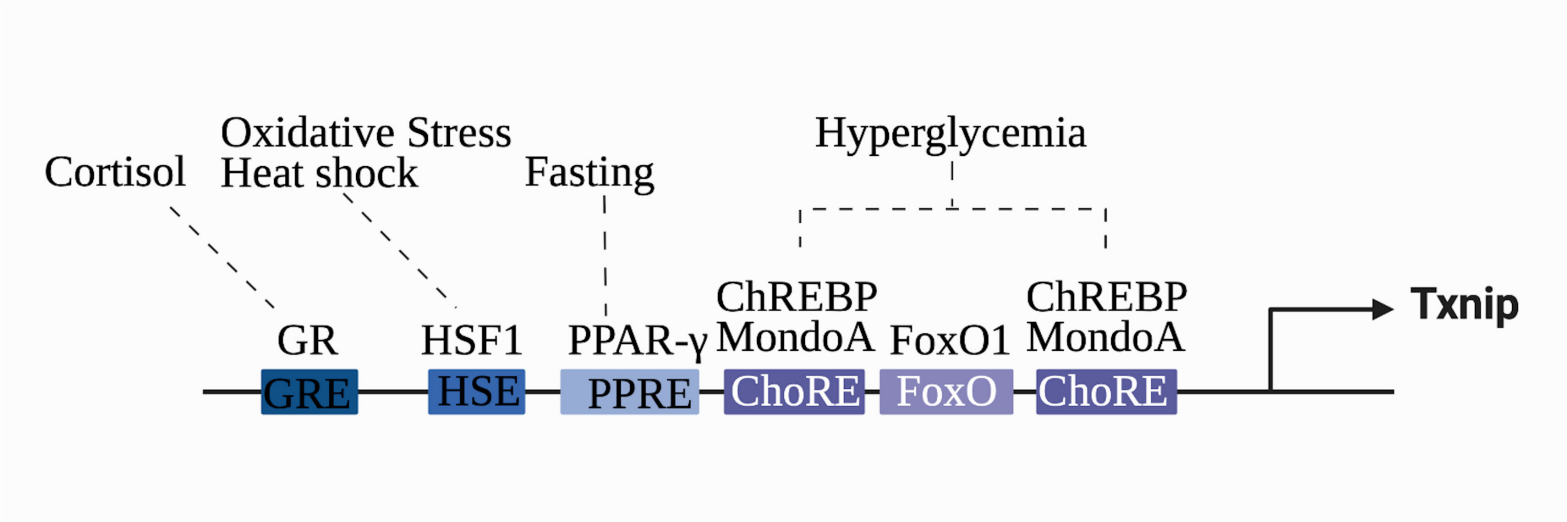
Transcriptional regulation of the *TXNIP* gene. *TXNIP* gene expression is regulated by various regulatory elements, transcription factors, and receptors. The different stimuli correspond to the respective transcriptional factors and binding sequences. Glucocorticoid-glucocorticoid responsive element (GRE), heat shock factor 1-heat shock element (HSE), peroxisome proliferator-activated receptor element (PPRE), carbohydrate-responsive element (ChoRE), and Forkhead Box O binding site (FOXO) are shown.

TXNIP gene expression is regulated by various regulatory elements, transcription factors, and receptors. Two E-box repeat motifs called the carbohydrate response elements (ChoREs), are located at 400 bp of the coding region of the TXNIP promoter and are characteristic of human TXNIP (22). ChoREs can be combined with the carbohydrate response element-binding protein (ChREBP) to enhance TXNIP responsiveness to glucose and carbohydrates (23). The ChREBP homologous protein MondoA can induce TXNIP mRNA expression (24, 25). Several transcription factors regulate TXNIP. Forkhead boxo1, a transcription factor highly enriched near the ChoREs promoter, competes with ChREBP for the TXNIP promoter to inhibit TXNIP transcription, which depends on the E-box repeat motif of the ChoREs (26). Another vital transcription factor is the heat shock factor. Its transcriptional activity is increased under stress; it binds to the heat shock factor element and mediates the transcription of endogenous TXNIP (27). In addition, the transcriptional levels of TXNIP are regulated by several receptors. TXNIP, also known as vitamin D3 upregulated protein 1, was initially identified in a study on the myeloid differentiation of HL-60 cells. TXNIP was upregulated in HL-60 cells treated with 1,25-dihydroxyvitamin D-3, and its cDNA was correspondingly expressed to produce a 46 kDa protein *in vitro* (28). However, it remains controversial whether the promoter region of TXNIP contains classical vitamin D receptor response elements (29). Evidence suggests that the CCAAT motif in the regulatory region of TXNIP is necessary for the vitamin D3 response (30). In addition, the glucocorticoid receptor (31) and peroxisome proliferator-activated receptor (32) are also associated with the transcription of TXNIP; thus, the gene transcription levels of TXNIP are affected by various regulatory mechanisms, and different response sites determine the corresponding stress conditions (**Figure 1**).

### 2.2 Structure of TXNIP

**Figure 2:**
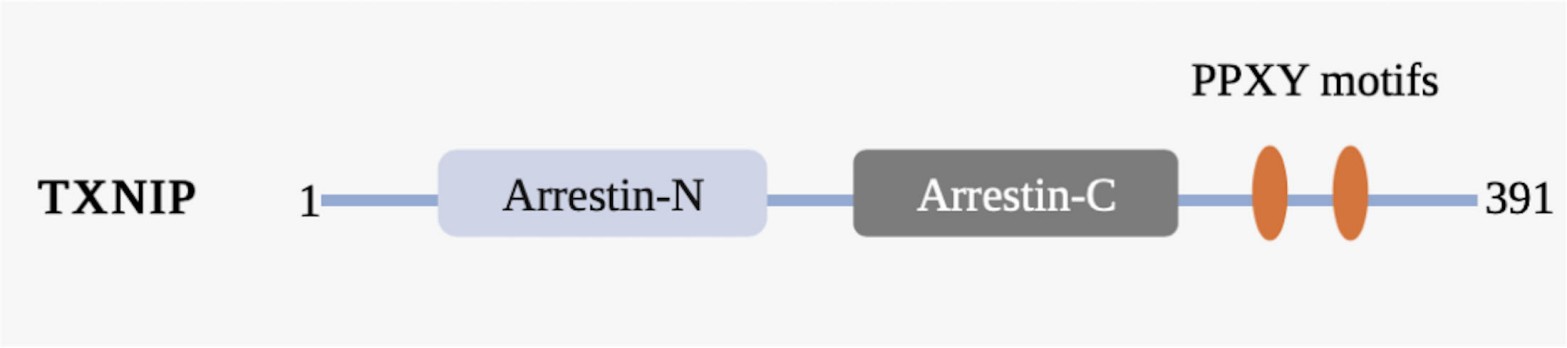
Schematic representation of the TXNIP domain organization. TXNIP contains amino acids 1−391. The α-arrestin TXNIP domain comprises a N-terminal domain (arrestin-N) and a C-terminal domain (arrestin-C), the C-terminal tail of which contains two PPxY motifs (yellow). Numbers indicate amino acids.

TXNIP is one of six α-arrestin proteins and is associated with five other α-arrestin proteins, four visual/β-arrestin proteins, and four vps26-related proteins, together constituting the arrestin protein family (33). Arrestin proteins can bind to photoactivated phosphorylated rhodopsin and inhibit (“arrest”) its ability to activate transduction proteins and phosphodiesterase (34). The polar core of charged residues in the N-domain of β-arrestins acts as a phosphate sensor and is the structural basis for the endocytosis mechanism involving the phosphorylated G protein-coupled receptor (35). TXNIP and five other proteins, known as arrestin domain-containing proteins 1–5 (ARRDC1–5), are referred to as α-arrestins based on phylogenetic studies. Although the arrestin N and C domains of α-arrestins are similar to those of β-arrestins, they are characterized by a highly conserved PPxY motif at the C-terminal tail, which can bind to the WW domains of proteins such as ubiquitin ligase (33). TXNIP has the same arrestin N and arrestin C domains and PPxY motif observed in other α-arrestins, enabling it to interact with other proteins and forming the structural basis for TXNIP to exert redox-independent functions (**Figure 2**).

### 2.3 Functions of TXNIP

#### 2.3.1 TXNIP and oxidative stress

**Figure 3:**
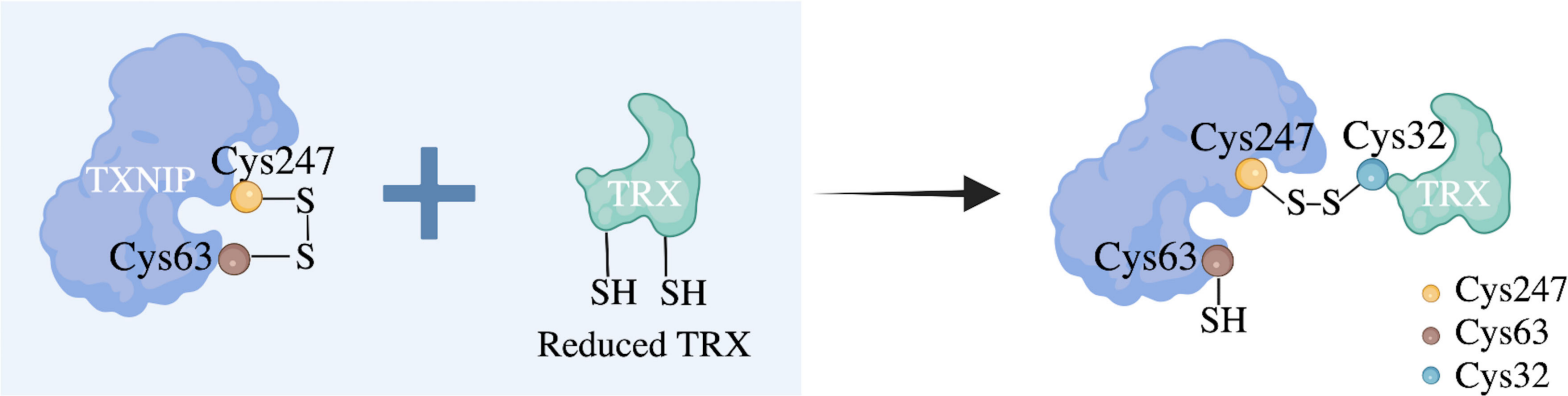
Proposed molecular mechanism of the negative regulation of TRX by TXNIP. The cellular redox state-dependent disulfide bond switching mechanism regulates the inhibition of thioredoxin (TRX) by thioredoxin-interacting protein (TXNIP). TXNIP contains an intramolecular disulfide band between cysteine residue (Cys) 63 and Cys247 that can interact effectively with TRX. Under normoxic conditions, TXNIP forms stable TRX-mixed disulfide bonds by disulfide exchange with reduced TRX and binds to TRX through the intermolecular disulfide bond formed between TXNIP Cys247 and TRX Cys32 to inhibit its reducing activity. Cys residues are indicated as dots.

TRX, an important cellular redox protein associated with cellular redox and energy metabolism, is located in both the mitochondria and cytoplasm and enables TXNIP to interact with TRX-1 in the cytoplasm and TRX-2 in the mitochondria. This suggests that TXNIP plays a role in both these cellular regions (36). Detailed 3D structural models of TXNIP have been reported; these include some surface residues and the unique arrestin structure domain, among which cysteine residue (Cys) 247 is the most widely examined (37). Studies have confirmed that the Cys247 on TXNIP forms stable mixed disulfide bonds with the Cys32 on TRX through disulfide bond exchange, and these characteristics differ from those of other arrestin family members (38). TRX1 can scavenge ROS, while TXNIP cysteine residues can directly bind to the active site of reduced TRX to form the TXNIP–TRX1 complex and inhibit the antioxidant capacity of TRX1 (39). Therefore, TXNIP is regarded as an endogenous negative regulator of TRX1 that regulates cellular redox reactions, exhibiting a TXNIP redox reaction-dependent function (**Figure 3**).

Notably, unlike other arrestin protein family members, TXNIP is the only α-arrestin protein that binds to TRX through two pivotal cysteines that are conserved (38). Therefore, TXNIP binds with TRX to form a TXNIP–TRX complex or interacts with other proteins through the characteristic α-arrestin protein structure to transmit intracellular signals. Previous studies have identified the redox-dependent and -independent functions of TXNIP using wild-type TXNIP and produced TXNIP C247S (TRX binding site) mutants that cannot bind TRX (40–42). It has been proposed that TRX regulates the lipid inhibition function of TXNIP by enhancing its stability, rather than TXNIP regulating TRX (41). TXNIP interacts with different cell substrates at different binding sites; the regulatory relationship between TXNIP and TRX differs in different cell types and under various physiological conditions.

However, TXNIP-deficient mice show little change in intracellular TRX1 activity following TXNIP deletion (43). These data suggest that although overexpression of TXNIP inhibits cytoplasmic TRX1 activation, TXNIP, at the physiological level, is localized to the nucleus and cannot enter the cytoplasm and bind to TRX1 to inhibit its activity. Under oxidative stress, the localization and function of TXNIP change, and TXNIP shuttles between the nucleus and cytoplasm. Upon intracellular ROS accumulation, TXNIP shuttles to the mitochondria, where it binds to and oxidizes TRX2 to form a TXNIP–TRX2 complex, which in turn inhibits the association of TRX2 with apoptotic signal-regulated kinase 1 (ASK1). This mediates the phosphorylation and activation of the ASK1 signal protein and induces the mitochondrial apoptosis pathway through cytochrome C release and caspase-3 cleavage, ultimately inducing apoptosis (44). Knockdown of TXNIP or the use of antioxidants significantly reduces TXNIP expression and oxidative stress levels (45, 46). The ROS/TXNIP pathway is also involved in the pathogenesis of several metabolic diseases such as intervertebral disc degeneration (47) and non-alcoholic fatty liver disease (48).

#### 2.3.2 TXNIP and pyroptosis

**Figure 4:**
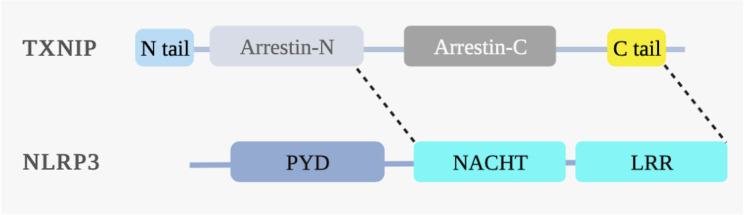
TXNIP binds to NLRP3 under ROS. ROS induces the interaction between the NLRP3 and TXNIP domains. The NLRP3 structural domains include the nucleotide-binding oligomerization domain (NACHT), pyrin domain (PYD), and leucine-rich repeat domain (LRR). The N-terminal arrestin (arrestin-N) domain of TXNIP interacts with the NACHT domain of NLRP3. The C-terminal carboxyl extension domain of TXNIP binds to the LRR domain of NLRP3, thereby activating the NLRP3 inflammasome under ROS.

In addition, TXNIP is essential for mediating cellular inflammatory responses, mainly those related to activating the intracellular Nod-like receptor protein 3 (NLRP3) inflammasome. When intracellular ROS levels increase excessively, TXNIP is separated from TRX, the arrestin-N domain of TXNIP interacts with the NACHT domain of NLRP3, and the C-terminus binds to the LRR domain of NLRP3, thereby activating the NLRP3 inflammasome (12). These observations illustrate that TXNIP functions through its α-arrestin protein domain **(** **Figure 4** **)**. Formation of the NLRP3 inflammasome complex activates caspase-1, followed by the maturation and release of the inflammatory cytokines IL-1β and IL-18. The process can also lead to a rapid form of proinflammatory cell death named “pyroptosis,” characterized by cell swelling, membrane rupture, plasma membrane pore formation, massive leakage of cytoplasmic contents, and high levels of inflammation (49). Gasdermin D is a crucial executor of pyroptosis and functions by releasing the N domain cleaved by caspase-1 (50). Results from *in vitro* experiments have shown that TXNIP is involved in the activation of the NLRP3 inflammasome pathway and the induction of inflammatory responses (51–54).

However, other findings suggest that TXNIP is dispensable in activating the NLRP3 inflammasome. Compared to wild-type mice, TXNIP-deficient mice showed no significant differences in IL-1β secretion from bone marrow derived macrophages in response to islet amyloid polypeptide or other inflammatory activators (55). In addition, nuclear factor-κB expression is strongly promoted by TRX1, but not by TXNIP, to bind to the DNA and activate the NLRP3 inflammasome. One study reported no defect in the secretion of IL-1β from TXNIP-deficient bone marrow derived macrophages compared to that from wild-type macrophages (56). Considering these results, the relationship between TXNIP and pyroptosis, particularly its interaction with NLRP3, requires further analysis.

#### 2.3.3 TXNIP and ferroptosis

Iron, an essential trace element in the human body, is involved in several biochemical processes. Excessive iron levels can disrupt redox homeostasis and elevate ROS levels, thereby exacerbating oxidative stress. Oxidative stress can in turn induce “ferroptosis,” a regulatory cell death that depends on iron and ROS. It is characterized by the excessive production of ROS caused by the downregulation of glutathione peroxidase 4 (GPx4) and cystine/glutamate antiporter system X_c_^−^, and is mediated by the Fenton reaction (57, 58). Studies have shown that TXNIP expression is altered under iron overload and may be associated with ferroptosis. In human breast cancer cells, the functional destruction of CDGSH iron sulfur domain 2 (privatization of autophagy factor-1) increases the expression of TXNIP, which is dependent on the accumulation of mitochondrial labile iron and on mitochondrial ROS levels. During this process, the expression levels of the ferroptosis marker GPx4 are reduced, whereas those of transferrin receptor and lipid peroxidation are increased (59). Meanwhile, one study showed that the protein expression of TXNIP in the heart tissue significantly decreased during iron overload regardless of oxidative stress, which is a possible protective mechanism during iron overload (60). In addition, TXNIP has been identified as one of the ferroptosis-related genes associated with bladder cancer (61). These findings suggest an association between TXNIP, iron, and ferroptosis, which requires further investigation.

Increasing evidence suggests a link between iron overload and bone diseases. *In vitro* findings have confirmed that iron overload induces ferroptosis and inhibits osteogenic differentiation and the mineralization of OBs (62–64). The diabetes microenvironment also enhances osteocyte ferroptosis, which is manifested by high lipid peroxidation, iron overload, and the abnormal activation of the ferroptosis pathway (65). Ferroptosis is a risk factor for OP (66, 67) and excessive iron can accelerate OA by inducing CC ferroptosis (68). However, an *in vivo* study showed that ferroptosis was declined in RA and RA fibroblast-like synoviocytes (69). Synovial hyperplasia causes cartilage damage during RA. The induction of ferroptosis in fibroblasts can slow the progression of arthritis. Ferroptosis reduced the number of synovium fibroblasts in a collagen-induced arthritis model (70). Although TXNIP is associated with iron overload and ferroptosis in other diseases, its role in the association between iron overload and bone disorders remains unclear.

## 3 TXNIP pathway in bone metabolic disorders

Previous studies have shown that the levels of glutathione, TRX, the major thiol antioxidants, and TRX reductase (the enzyme responsible for reducing TRX) significantly decrease in the bone marrow of ovariectomized rodents and rapidly return to normal upon supplementation with exogenous estrogen. This suggests that estrogen deficiency leads to bone loss by reducing thiol antioxidants (71). *In vivo* findings showed that the overexpression of TRX in TRX-transgenic mice partially restored the reduced bone mineral density and prevented streptozotocin-induced bone formation and osteopenia in diabetes (72). Recently, TXNIP has been reported to be associated with OP. Patients with endogenous Cushing’s syndrome (CS) exhibit serious systemic manifestations, including acquired OP and fractures, caused by the long-term administration of glucocorticoids. Lekva et al. (73) analyzed the whole-genome expression profile of bone biopsy specimens from patients with CS and detected the upregulation of *TXNIP*. This gene is downregulated in patients following surgical intervention. TXNIP silencing can increase the number of OBs and the expression of the bone formation marker osteocalcin (OCN), suggesting that TXNIP mainly inhibits OBs and bone formation functions to accelerate glucocorticoid-induced OP. *In vivo* studies in glucocorticoid-induced OP rats showed that the expression of TXNIP in the serum and bone is increased, suggesting that TXNIP is, to some extent, associated with the pathogenesis of glucocorticoid-induced OP (74). Goto-Kakizaki (GK) rats, a model of type 2 diabetes mellitus (T2DM), have significantly reduced bone mass, although the expression of TXNIP in the bone tissue is significantly increased. Simultaneously, in this model, bone formation-related parameters, such as OCN expression, are significantly reduced, whereas the levels of bone resorption markers, such as tartrate-resistant acid phosphatase (TRAP), are significantly increased. Moreover, calcitriol treatment reduces MDA and TXNIP expression in GK rats, suggesting that TXNIP-mediated oxidative stress reduces bone quality in these rats (16). In rats, oral administration of aluminum for 12 weeks significantly upregulated the expression of TXNIP protein in the femur, activated the NLRP3 inflammasome pathway, and increased the serum levels of IL-1β and IL-18, resulting in bone loss (20). These findings indicate that TXNIP aggravates bone destruction by mediating inflammation. TXNIP knockout (KO) mice have been used to determine the role of TXNIP in glucocorticoid OP. The experimental data suggested that only certain indicators differed; the bone formation type I procollagen amino-terminal peptide and bone absorption type I collagen carboxyl-terminal peptide indices in the sera of TXNIP KO mice were lower than those in the sera of wild-type mice. However, compared with that in the TXNIP KO group treated with prednisone acetate, the authors observed no significant difference in the bone biomechanical parameters and parameters measured *via* micro-computed tomography in the untreated TXNIP KO group (74). Therefore, there was not sufficient evidence to suggest that TXNIP KO rescued the bone loss caused by glucocorticoid.

OA is a degenerative disease of the joints that causes chronic pain, cartilage degeneration, synovitis, and even disability. OA progression is accompanied by cartilage wear, subchondral bone sclerosis, osteophyte formation, and inflammatory effusion. Cartilage and bone metabolism are involved in cartilage layer wear and subchondral osteophyte formation (75). TXNIP affects the aging process in different tissue types by maintaining the redox state. *In vivo* and *in vitro* evidence has shown that TXNIP expression is increased in elderly *Drosophila* senescence and primary cells (including peripheral blood T cells and non-hematopoietic cells) and TXNIP overexpression significantly shortens the lifespan of *Drosophila* (76). Sirtuin 6 (SIRT6) is also a key regulator of longevity and drives the aging process. SIRT6 transgene overexpression has been shown to improve metabolic function and prolong the lifespan of mice. Increased TXNIP expression during aging is associated with reduced SIRT6 activity. Additionally, the number of TXNIP-positive CCs in the articular cartilage tissues of the elderly is significantly higher than that in young individuals (77). Therefore, TXNIP accelerates CC dysfunction and aging. TXNIP regulates CC development and affects articular tissue homeostasis. TXNIP staining was significantly enhanced in the CCs of mice with surgically induced post-traumatic OA (instability in the medial meniscus) (78). However, the role of TXNIP in OA remains unclear. Another study confirmed that TXNIP mRNA and protein are expressed in the articular cartilage of normal human donors, but their expression is significantly reduced in OA (79). This suggests that TXNIP plays different roles at different ages or stages of the disease, which warrants further investigation.

RA is a heterogeneous systemic autoimmune disease characterized by chronic synovial inflammation and joint structural damage. In addition, bone complications, which are the main extra-articular complications in patients with RA, include three main types: periarticular bone loss, bone erosion, and systemic OP (80). Several factors lead to bone destruction and OP in patients with RA, such as inflammation, the deterioration of cortical bone quality, adverse reactions to drugs used to treat RA (such as glucocorticoids), hypokinesia, and nutritional effects (such as vitamin D deficiency) (81). Genome-wide expression analysis has shown that TXNIP is one of 110 RA-related genes in human CCs (82). Stimulating human CCs with supernatant from the synovial fibroblasts of patients with RA reduced their *TXNIP* gene levels. These levels were increased following treatment with methotrexate, diclofenac, and prednisolone; however, the degree of increase differed (82). In contrast, in fibroblast-like synovial cells from adjuvant arthritis, the expression of NLRP3 inflammasome (including NLRP3, ASC, and caspase-1) was significantly upregulated *in vitro*. Silencing TXNIP RNA significantly inhibited the formation of the NLRP3 inflammasome and the secretion of IL-1β and MMP-1 (83). Furthermore, miRNAs targeted TXNIP. In fibroblast-like synovial cells, luciferase analysis showed that the 3′UTR of TXNIP mRNA in rats was targeted by miR-20a, while miR-20a overexpression reduced the expression of TXNIP, thereby inhibiting the formation of NLRP3 inflammasome and MMP-1 (83). Another animal study confirmed that the expression levels of TXNIP, NLRP3, ASC, and caspase-1 in the synovia of adjuvant arthritis rats were significantly increased (18). Macrophages secrete proinflammatory cytokines and chemokines such as TNF and IL-1β, leading to joint pain and injury. In contrast, macrophages absorb anti-inflammatory cytokines to negatively regulate autoimmune activities and protect the joint tissue. The plasticity of macrophages in RA pathogenesis is known as “polarization.” TXNIP in macrophages from patients with RA is regulated by miR497 and CDKN2B antisense RNA1, a long non-coding RNA that targets miR-497. This regulatory axis promotes M1 polarization but inhibits M2 polarization, resulting in the inflammatory activity of macrophages. In other words, TXNIP promotes the expression of proinflammatory factors such as TNF and IL-1β but inhibits the expression of anti-inflammatory factors by affecting macrophage polarization, ultimately leading to RA (84). Therefore, TXNIP mediates bone destruction in RA by controlling the secretion of inflammatory factors. Further investigations are warranted to determine the exact role of TXNIP in RA.

## 4 Mechanisms underlying TXNIP involvement in bone metabolism

### 4.1 TXNIP and osteoblasts

TXNIP is activated *via* glucocorticoid stimulation and contains a classical glucocorticoid binding site. *In vitro* experiments have shown that TXNIP mRNA and protein levels can be overactivated by dexamethasone and trigger bone loss by upregulating the mitochondrial oxidative phosphorylation pathway in MG63 cells (74). *In vivo* experiments also found that glucocorticoids tightly regulate TXNIP expression in bones in CS, while the TXNIP gene in bone tissue is downregulated following surgical treatment for CS. In addition, *in vivo* data indicate a correlation between TRX and TXNIP, while *in vitro* data showed that TXNIP mRNA and protein levels were upregulated during human fetal OB maturation and induced by dexamethasone stimulation. Silencing TXNIP in OBs increases OCN and alkaline phosphatase (ALP) activities, illustrating the adverse effect of TXNIP-mediated glucocorticoid on OB differentiation (73). Lekva et al. (85) further verified that TXNIP in human fetal OB may affect glucose metabolism and insulin resistance by influencing insulin signaling in OBs. TXNIP siRNA enhances OCN secretion and increases the expression of insulin receptors in OBs. An TXNIP siRNA-treated conditioned medium from OBs also promoted insulin secretion and reduced inflammatory responses in human islet cells. These findings indicate that TXNIP expression is regulated by glucocorticoids and affects glucose metabolism. In addition, TXNIP adversely affects OBs by inducing the inflammasome pathways. NLRP3 inflammasome activation has been confirmed in abnormal bone development, and inhibiting NLRP3 inflammasome activation can reduce bone loss (86). Aluminum stimulates activation of the NLRP3 inflammatory pathway and increases TXNIP expression in MC3T3-E1 osteoblastic cells. However, TXNIP siRNA reduces the aluminum-induced expression of NLRP3 and the inflammatory cytokines IL-1β and IL-18 and increases OCN and ALP levels. This suggests that TXNIP improves aluminum-induced bone loss and formation by inhibiting inflammation (20). TXNIP is regulated by endogenous non-coding RNA and plays a key role in OBs. The long non-coding RNAs MEG3 and TXNIP are significantly upregulated in OP rats, whereas microRNA (miRNA-214) expression is downregulated. Dual-luciferase reporter gene detection results have confirmed that *TXNIP* is the downstream target gene of miRNA-214, and MEG3 silencing and miRNA-214 overexpression promote OB proliferation and differentiation by downregulating TXNIP and increasing osteoprotegerin (OPG) expression (87).

### 4.2 TXNIP and osteoclasts

TXNIP is a crucial regulator of OC proliferation and differentiation. TXNIP expression is reduced during human OC differentiation induced by soluble nuclear factor-κB ligand-receptor activator (sRANKL) and macrophage colony-stimulating factor (M-CSF). However, the activity of TRX-1 increases, while the antioxidant N-acetylcysteine reverses this pattern and significantly inhibits OCs (88). Interestingly, *in vitro* findings have shown that TXNIP silencing increases the RANKL/OPG ratio. Specifically, TXNIP upregulation increases OPG and reduces RANKL expression, thereby suppressing OC formation under the indirect effect of OBs during bone conversion. Moreover, conditioned media derived from OBs in which TXNIP was silenced were treated with human OC precursors, and the results showed that the activity of TRAP, a marker of OC differentiation, was significantly increased following treatment (73). Another study showed that TXNIP protein expression did not differ with or without RANKL stimulation, whereas the expression of TXNIP in OCs was significantly increased when combined with D-allose during the differentiation of the rat Raw264 OC cell line, as was the expression of TRX. Moreover, TXNIP overexpression inhibited the formation of TRAP-positive cells and OCs, suggesting that the rare sugar D-allose negatively regulates OC differentiation through TXNIP upregulation (89). Therefore, TXNIP overexpression negatively affects OC differentiation, which is associated with the molecular regulatory mechanisms between TXNIP and TRX induced by RANKL. This requires further study.

### 4.3 TXNIP and chondrocytes

Aging is associated with OA and mainly involves defects in autophagy. Under physiological stress, TXNIP is upregulated to promote autophagosome maturation (90). *In vitro* studies have shown that the combined deletion of TXNIP and Redd1 in human primary CCs leads to autophagy defects, suggesting that the Redd1/TXNIP complex is necessary for inducing autophagy in CCs (79). Mammalian target of rapamycin (mTOR) is a highly conserved serine-threonine kinase overexpressed in cancer. TXNIP interacts with Redd1 at the N-terminus to stabilize it, thereby inhibiting mTOR activity (91). However, in myocardial ischemia/reperfusion injury, myocardial-specific TXNIP is overexpressed and directly interacts with the autophagy regulatory factor Redd to inhibit mTOR, thereby aggravating cardiac ischemia/reperfusion injury (92). Notably, TXNIP may play an inhibitory role in regulating autophagy in metabolic and degenerative diseases. For example, in the pathogenesis of Parkinson’s disease, TXNIP inhibits the autophagy flux-induced accumulation of alpha-synuclein (93). Animal studies have also confirmed that the expression of TRX family members and anti-aging protein Klotho is reduced and induces apoptosis and IL-1β release in the articular cartilage of OA mice. These effects are reversed by the overexpression of Klotho *via* blockade of TXNIP and pyroptosis-related molecules, such as NLRP3 and caspase-1 (94). In an OA CC model, IL-1β induced the expression of the proinflammatory factors TNF-α, IL-1β, and IL-6 in the human chondrosarcoma cell line SW1353 and normal human CCs (C28/I2), which was partially reversed by TXNIP overexpression (95). The expression of SIRT6, a key regulator of aging, was significantly reduced in CCs isolated from samples obtained from elderly individuals, but overexpression of SIRT6 specifically increased the basal levels of the antioxidant protein TRX, whereas TXNIP levels were downregulated. In contrast, Sirt6 deletion reversed these effects, and TXNIP levels were upregulated in CCs derived from SIRT6 KO mice (77). These studies indicate that TXNIP participates in the normal metabolism of CCs by regulating autophagy. However, abnormal TXNIP expression, or that driven by various aging factors, disturbs the metabolism of CCs and degenerates articular cartilage in specific body stages, especially during the processes of aging and inflammation, eventually influencing the pathogenesis of OA.

**Figure 5:**
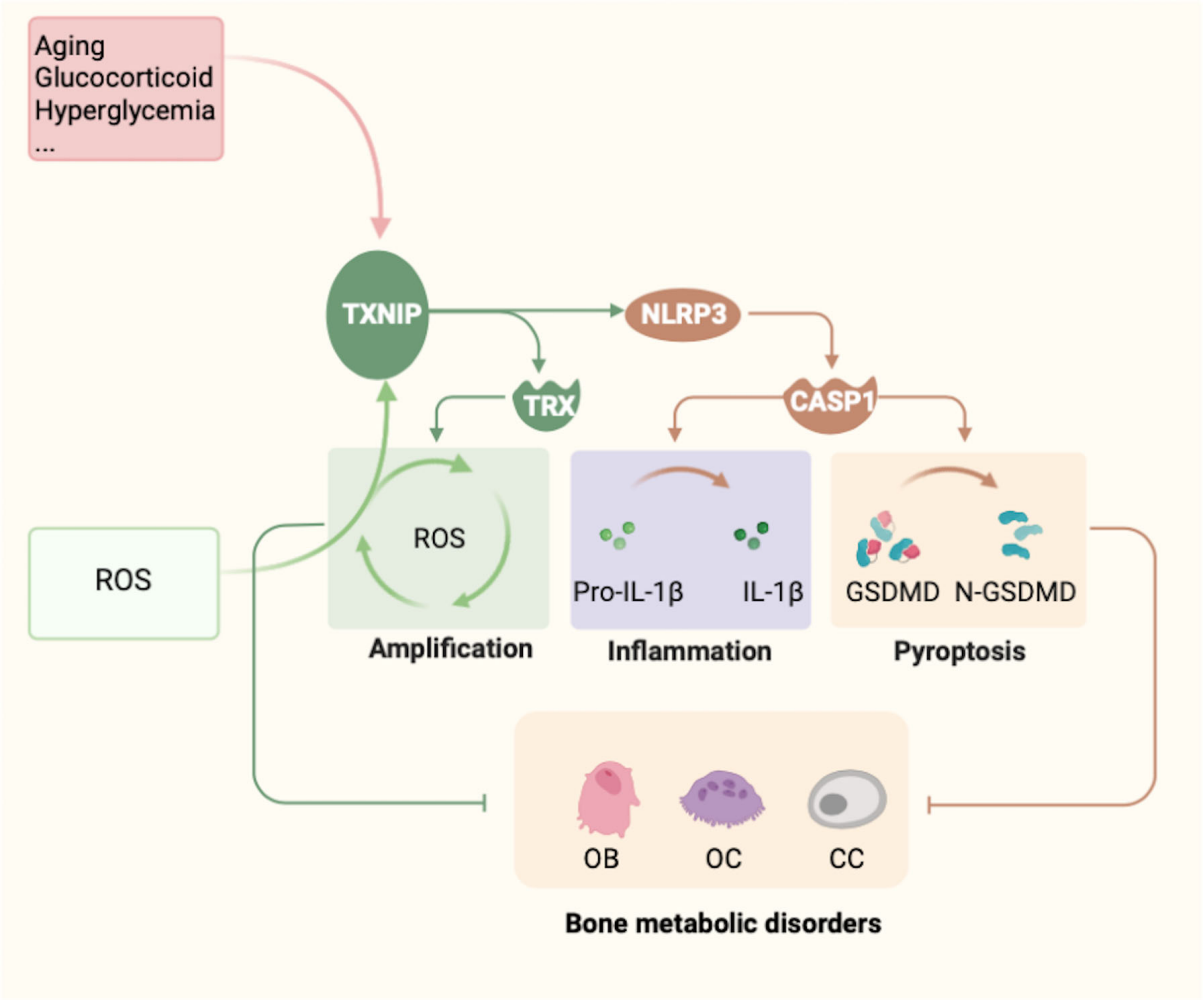
Summary of TXNIP regulation and functions. Several factors, such as glucose, glucocorticoids, metals, and aging, are known to regulate the expression of TXNIP. In contrast, TXNIP induces the emergence of oxidative stress, leading to the release of a large amount of ROS and amplifying the body’s oxidative stress response. However, owing to changes in the expression of TXNIP, three main effects can be noted: (1) The formation and function of the TXNIP–TRX complex. (2) TXNIP functions as a potential binding partner of NLRP3. TXNIP dissociates from TRX and binds to NLRP3, resulting in activation of the NLRP3 inflammasome pathway under ROS. Thus, caspase-1 is activated and promotes the maturation and release of the inflammatory factors IL-1β and IL-18, which eventually leads to cellular inflammation. (3) The mechanism by which TXNIP and NLRP3 form a complex to induce the pyroptosis-related factor Gasdermin D remains to be determined. Changes in these three mechanisms are the possible underlying mechanisms of TXNIP causing the abnormal metabolism of bone cells, mainly osteoblasts, osteoclasts, and chondrocytes, ultimately leading to bone metabolism disorders.

In conclusion, the relationship between TXNIP and bone cell differentiation processes is complex. The normal maturation and differentiation of OBs and OCs require the basic regulation of TXNIP in their physiological processes, demonstrating the importance of TXNIP in bone metabolism. However, existing data show that TXNIP is closely related to bone metabolism and that abnormal TXNIP expression affects the differentiation of OBs, OCs, and CCs by affecting intracellular redox homeostasis and inflammation. Finally, TXNIP overexpression or loss affects the normal metabolism and function of these cells which are associated with bone metabolism disorders **(** **Figure 5** **).** Although TXNIP has a limiting effect on OCs through indirect effects caused by intercellular paracrine signaling, this effect is limited and does not suggest that TXNIP is entirely beneficial in bone resorption. Under most pathological conditions, the direct influence of disease and stress on OCs is dominant, while TXNIP can only be regarded as one of the factors involved in the metabolic process. In addition, studies are needed to determine the role of TXNIP and other regulatory factors in bone metabolism.

## 5 TXNIP is a potential therapeutic target for bone metabolism disorders

### 5.1 Proposed TXNIP inhibitors

TXNIP has attracted attention in drug development owing to its multiple functions and involvement in metabolic disorders, inflammatory diseases, neurodegenerative diseases, and cancer. TXNIP plays a tumor inhibitory role as an apoptosis inducer; thus, TXNIP agonists may contribute to anticancer treatment (96, 97). In contrast, a large amount of data strongly supports that TXNIP inhibition can be used to treat metabolic disorders and related diseases (13, 98). Currently, there are no TXNIP inhibitors available, and only a few specific TXNIP inhibitors are in the clinical stage. TXNIP expression is regulated by a unique E-box motif in the TXNIP promoter, especially in the case of high glucose levels and diabetes. The small-molecule compound SRI-37330 inhibits the expression of *TXNIP* in human islets through this element. Unlike *TXNIP*, *TRX* and other arrestin genes such as *ARRB1* and *ARRDC3* are not affected by SRI-37330, whereas inflammasome NLRP1 is downregulated (99). Ahn et al. (100) used high-throughput chemical and biological screening to identify a small molecular probe, SBI-477, that can cooperate with triglycerides to enhance basal glucose uptake in skeletal muscle cells. SBI-477 stimulates the insulin pathway by inactivating the MondoA transcription factor. Thus, the protein expression of the insulin pathway inhibitors TXNIP and ARRDC4 decreased and improved insulin resistance and lipotoxicity (100). The two proposed TXNIP small-molecule inhibitors exert their effects mainly *via* the transcription of TXNIP, and SRI-37330 has a specific role in inhibiting TXNIP. However, these inhibitors may be cell- or tissue-dependent. For example, in hepatocytes, SRI-37330 affects the function of glucagon in various ways unrelated to its expression (99). In addition, some inhibitors regulate the TXNIP effect through intermolecular interactions. Peptides derived from the TXNIP–p38 interaction motif, 13 amino acid-containing peptides (TN13), inhibit the TXNIP–p38 interaction and significantly restore aging hematopoietic stem cells (101). Some phytochemicals can exert powerful anti-oxidation and anti-inflammatory effects by inhibiting the combination of TXNIP and NLRP3 and have significant beneficial effects on inflammation, cancer, diabetes, and other diseases. For example, ruscogenin reduces the blood-brain barrier dysfunction caused by cerebral ischemia by inhibiting TXNIP/NLRP3 inflammatory corpuscle activation and the mitogen-activated protein kinase K pathway (102). Resveratrol reduced the expression of the TXNIP/TRX/NLRP3 signaling pathway, suppressing amyloid-β-induced microglia inflammation (103). Doxofylline, a theophylline derivative, can also inhibit the production of mitochondrial ROS induced by lipopolysaccharides and alleviate epithelial inflammation by improving various cellular pathways, including TXNIP–NLRP3 inflammatory activation (104). However, the exact regulatory mechanisms of TXNIP in these phytochemicals are not well understood.

### 5.2 Application and mechanism of TXNIP regulators in bone metabolism disease

The role of TXNIP in bone metabolism has been confirmed, hence TXNIP inhibitors in bone metabolism have been developed as a treatment strategy as shown in (**Table 1**). As described above, TN13, a TXNIP inhibitor, targets the TXNIP–p38 docking motif. Binding of TN13 to the human immunodeficiency virus TAT transduction domain sequence at the N domain, known as TAT-TN1, decreases RANKL-stimulated osteoclastogenesis and bone resorption by inhibiting the p38/NF-κB/NFATc1 signaling pathway and reducing bone loss in ovariectomized mice (15). T2DM induces bone loss in GK rats. Treatment with 1,25-dihydroxy vitamin D3 significantly reduces T2DM-induced bone loss, improves bone microstructure and biomechanical properties, and reduces serum glucose and glycosylated serum protein levels. Meanwhile, TXNIP expression in the bone decreases, whereas bone formation biomarkers increase significantly (16). A previous study showed that tanshinol improved microcirculation disorder and damaged bone formation in rats by inhibiting the activation of the TXNIP signaling pathway in glucocorticoid-induced OP rats and human OB-like cells (MG63), reversing the downregulation of the Wnt and vascular endothelial growth factor pathways (17). Nicotinamide mononucleotide, a biosynthetic intermediate of nicotinamide adenine dinucleotide, improved aluminum-induced bone injury by inhibiting the TXNIP–NLRP3 inflammasome pathway (20). Another *in vivo* study showed that a “Sanse Powder” essential oil nanoemulsion reduced the expression of the ERS/TXNIP/NLRP3 signaling pathway and the levels of the inflammatory mediators IL-1β and IL-18, reducing anti-synovial inflammation and treating OA (19). Loratadine inhibits the expression of TXNIP and NLRP3 inflammasome and related components, including NLRP3, ASC, and cleaved caspase-1, by inhibiting the production of mitochondrial ROS and NADPH oxidase subunit NOX4 to alleviate SW1353 CC injury induced by advanced glycation end products. In addition, loratadine inhibits the expression of Nrf2. The silencing of Nrf2 expression eliminates the inhibitory effect of loratadine on NLRP3 inflammasome activation. Therefore, loratadine protects CCs from AGE-induced TXNIP/NLRP3 inflammasome activation by regulating the expression of the transcription factor Nrf2, thereby alleviating OA (21). The expression levels of TXNIP, NLRP3, ASC, caspase-1, and NF-κB significantly increased in the synovium of an adjuvant arthritis rat model but significantly decreased after combined treatment with atorvastatin and quercetin. Simultaneously, arthritis-related inflammation and oxidative stress parameters in the serum were reduced (18). In conclusion, the application of specific TXNIP inhibitors in bone metabolic diseases has not been reported, although some traditional Chinese medicines and approved drugs have been used. The mechanisms underlying the specific effects between these drugs and TXNIP require further exploration.

**Table 1:** Application of TXNIP regulators in bone metabolic diseases.

Drug name	Mechanism	Disease	Status	Reference
TAT-TN13	TXNIP–p38	Postmenopausal OP	*In vivo*	(15)
1,25-Dihydroxy vitamin D3	TXNIP	Type II diabetes-induced OP	*In vivo*	(16)
Tanshinol	TXNIP, Wnt	Glucocorticoid- induced OP	*In vivo*	(17)
Atorvastatin/Quercetin	TXNIP/NLRP3	RA	*In vivo*	(18)
“Sanse Powder” essential oil nanoemulsion	ERS/TXNIP/NLRP3	OA	*In vivo*	(19)
Nicotinamide mononucleotide	TXNIP/NLRP3	Aluminum-induced OP	*In vivo*	(20)
Loratadine	TXNIP/NLRP3, ROS, Nrf2	OA	*In vitro*	(21)

## 6 Summary and prospects

We described the direct role of TXNIP, an α-arrestin protein that specifically binds to TRX, in several bone metabolic diseases and the underlying mechanisms. TXNIP affects OBs, OCs, and CCs by influencing intracellular redox homeostasis and regulating inflammation. Moreover, TXNIP can be used to predict several bone metabolic diseases, including OP, OA, and RA. TXNIP affects bone metabolism through redox-dependent and -independent pathways, which should be distinguished as this may help to explain the specific metabolic functions and regulatory mechanisms of TXNIP in more detail. Finally, TXNIP-targeted therapy for OP may be an effective treatment strategy for preventing OP by increasing OB proliferation and differentiation or OC function and integrity. Other chronic inflammatory bone diseases, such as OA and RA, require the specific targeting of bone cells. However, as the role of TXNIP in the bone metabolism of these cells is complex, it is necessary to conduct TXNIP KO animal experiments for further insights. TXNIP is not only an inhibitor of TRX but also an important factor in glucose metabolism; TXNIP mutation in mice leads to impaired glucose homeostasis (105). Additionally, the expression of TXNIP in cancer is low and TXNIP overexpression inhibits the proliferation of cancer cells; therefore, it is regarded as a potential tumor suppressor. The risk of cancer development is high in TXNIP-deficient mice (106), which should be considered when knocking out TXNIP in bone research. Considering the necessity and multiple roles of TXNIP in bone cells, targeting molecules or signaling pathways that interact with TXNIP is a potential strategy for treating and preventing bone metabolic disorders. Thus, further preclinical and clinical investigations are essential for understanding TXNIP-specific inhibitors and developing new promising treatments to mitigate the health problems associated with bone metabolic disorders.

## Author contributions

NJ drafted and prepared the manuscript. NJ, JL, and CG reviewed and edited the manuscript. JA and CM extracted data and constructed the figures with software. XT considered the ideas and overall structure of the article. All authors read and approved the final manuscript.

## Funding

This work was supported by the National Natural Science Foundation of China under grant no. 81370970, and the Science and Technology Support Program of Gansu Province under grant no. 144FKCA075, the excellent postgraduate "Innovation star" Program of Gansu Educational Committee under grant no. 2021CXZX-046, the Gansu Natural Science Foundation Youth Project under grant no. 20JR10RA712.

## Conflict of interest

The authors declare that the research was conducted in the absence of any commercial or financial relationships that could be construed as a potential conflict of interest.

## Publisher’s note

All claims expressed in this article are solely those of the authors and do not necessarily represent those of their affiliated organizations, or those of the publisher, the editors and the reviewers. Any product that may be evaluated in this article, or claim that may be made by its manufacturer, is not guaranteed or endorsed by the publisher.
